# Our New York Letter

**Published:** 1886-11

**Authors:** 


### OUR NEW YORK LETTER.

TREATMENT OF TYPHOID FEVER IN THE NEW YORK HOSPITALS—DR. SPITZKA'S EXPERIMENTS IN THE PRODUCTION OF HYDROPHOBIA—A SUCCESSFUL CASE OF LAPAROTOMY IN CASE OF GUN-SHOT WOUND—TREATMENT OF VARICOCELE, ETC.

Thinking it might be interesting to ascertain some of the different methods of treating typhoid fever, at present in New York, and the results obtained, I visited some of the larger hospitals, saw the yearly reports and inquired regarding their mode of treatment. At the New York Hospital calomel is occasionally given early in the disease if constipation exists. This condition is afterwards relieved if necessary by enemata. Antipyrine is given in drachm doses by the rectum, and repeated every three hours if required. It is not administered with stimulants, and no cases of collapse have occurred from its use. Sponging and the bath are also resorted to. During the year the death rate from this disease was twenty per cent. At St. Vincent's Hospital no cathartics are given in typhoid. To reduce temperature quinine is administered in doses of 10 to 20 grains. Here they do not give antipyrine, or employ sponging, wet packing or baths. The mortality is forty per cent. At the Roosevelt Hospital calomel is occasionally given early in the constipated cases. Antipyrine is used and cold water applied to the surface by the different methods. Constipation during the course of the disease is relieved by an enema every third day. Ten per cent. of the cases died. At St. Francis Hospital calomel is frequently given during the early part of the fever. Antipyrine has been used in large doses, but 15 grains is now considered enough at once. Cold sponging is also resorted to. The death rate here was seventeen per cent. Cathartics are not given at the Presbyterian Hospital. Antipyrine is used either in 15 grain doses every three hours, or 30 grain doses when the temperature rises above 103° F. The bath and Kibbee cot are occasionally employed. For constipation enemata are administered. The death rate was twenty per cent. At the Mount Sinai Hospital Catholics are omitted in this fever. Antipyrine is largely used. Local application of cold water is rarely made. Here the death rate during the year was 11 per cent. In the German Hospital from 15 to 20 grains of calomel are always given if the patient be admitted before the end of the second week. After this a daily enema is administered if constipation is present. Antipyrine is always accompanied with stimulants, and in two patients taking half drachm doses temporary collapse occurred. Musk in 7 grain doses is considered here a brilliant heart stimulant, and is used three or four times daily in severe cases. Camphor ether is also used for the same object. The mortality was twelve per cent. In all these hospitals milk is the principal food in typhoid fever, and stimulants enter largely into the treatment of nearly all cases.

The experiments that Dr. Edward C. Spitzka has been conducting at the Veterinary Infirmary in East Fourteenth street, in support of his theory that rabies or hydrophobia can be produced in dogs by the insertion into their brains of any foreign substance, have satisfied him, so he says, of its truth.

Of six healthy dogs inoculated, five have died. Some portions of the spinal cord of a healthy calf and other substances were introduced into their brains. A mongrel Spitz dog, upon which had been used the weakest substance, was the fifth subject to succumb. If he had been on the street people would have fled from him. With a portion of this dead dog's brain, Dr. Spitzka inoculated a black and tan and will treat three more dogs at intervals of two days. Dr. Spitzka's experiments, if successful throughout, will show, it is claimed, the truth of the recently advanced idea that specific diseases will result from the action of foreign substance upon specific portions of the anatomy.

Dr. W. T. Bull has again performed a successful laparotomy in a case of gun-shot wound of the abdomen. A man twenty-five years old was shot in the abdomen, the bullet penetrating two inches below and to the left of the umbilicus. About two hours later at the Chambers Street Hospital, the abdominal cavity was opened, the wound having been previously explored and found to be a penetrating one. The patient showed no symptoms of intra-abdominal injury, the pulse being 94, respiration 24 and temperature 98° F. There was considerable blood in the cavity and the small intestine was perforated in two places. There was also a perforation of the sigmoid meso-colon, which furnished a profuse venous hemorrhage. The operation consumed one hour and a half and the patient stood it remarkably well. Since then no bad symptoms have arisen, no vomiting, very slight pain and some tympanitis. The pulse has not exceeded 110 and the temperature has not gone above 100° F. On the fifth day there was a natural movement of the bowels. The occurrence of a small mural abscess was the only adverse incident.

Some months ago Dr. E. L. Keys published an article on the treatment of varicocele and advocated the subcutaneous use of catgut. He used a needle threaded with a silk loop and a piece of catgut. The veins were pushed aside and the needle thrust from before backwards through the scrotum and made to emerge posteriorly, leaving the veins on the outer side of the needle. The catgut ligature was then pulled out of the eye of the needle and left hanging from the posterior wound. The point of the needle was then withdrawn within the scrotum. The veins were then allowed to join the rest of the spermatie cord, but the point of the needle was not withdrawn outside the anterior puncture in the scrotum. When the veins had passed internally to the point of the needle, the latter was manipulated around externally to the veins and made to emerge at the posterior puncture. The free end of the catgut was drawn through the silk loop and the needle rapidly withdrawn. After this the catgut was tied and cut short. Recently, while in England, Dr. Keys noticed that Mr. Lawson Tait used silk for most all purposes. The latter gentleman assured him that these ligatures at the bottom of wounds disappear without causing any trouble. Mr. Tait seemed to simply employ hot water in preparing his silk. On Dr. Keys, return to New York he operated for the first time with ordinary twisted silk prepared by boiling water. The patient had no bad symptoms and left the hospital on the sixth day. He has since used silk in five cases and all went well. Dr. Keys does not know whether the silk is absorbed or not, but a small lump remains about the cord and gradually diminishes. He believes it possible for the vein to again become pervious after the use of catgut, but not when silk is used. A. F.

*New York.*

